# Genetic background shapes the naïve IgM B cell receptor repertoire in mice

**DOI:** 10.3389/fimmu.2026.1890174

**Published:** 2026-09-22

**Authors:** Hina Wakimizu, Makoto Tsuiji, Chiaki Nishihara, Makoto Hattori, Tadashi Yoshida

**Affiliations:** 1 Department of Applied Biological Science, Tokyo University of Agriculture and Technology, Tokyo, Japan; 2 Department of Microbiology, Hoshi University School of Pharmacy and Pharmaceutical Sciences, Tokyo, Japan

**Keywords:** BCR repertoire, mouse strain, naïve B cell, V segment, VH/Vκ pairing

## Abstract

**Introduction:**

The B cell receptor (BCR) repertoire is generated through stochastic V(D)J recombination and further shaped by selection processes. Still, the influence of genetic background on the naïve repertoire remains unclear. In this study, we investigated inter-individual and inter-strain variations in the IgM BCR repertoire using naïve mice from three strains: BALB/c, C57BL/6N, and NC/Nga.

**Methods:**

Splenic IgM-expressing B cells were isolated at the single-cell level, and paired IGHV and IGKV sequences were analyzed.

**Results:**

Analysis of V gene usage revealed no significant differences in overall diversity among strains. However, analysis using the Jaccard index demonstrated that IGHV repertoires were highly similar within strains but apparently different between strains, whereas IGKV repertoires showed minimal variation. t-SNE and MDS visualizations further supported these findings, with clear clustering by strain observed for IGHV but not for IGKV. The CDR3 nucleotide and amino acid lengths showed no substantial differences among strains, and minimal sharing of CDR3 sequences was observed both inter- and intra-strain, indicating high individual variability. Notably, analysis of IGHV–IGKV pairing revealed that each VH segment was preferentially associated with a specific Vκ segment in a strain-dependent manner.

**Discussion:**

These results suggest that the naïve BCR repertoire is influenced by genetic background, particularly through biases in heavy-chain gene selection and VH/Vκ pairing. This intrinsic repertoire bias may contribute to strain-specific differences in antigen recognition and susceptibility to some immune-related diseases.

## 1 Introduction

Immunoglobulin (Ig) is the soluble form of the B cell receptor (BCR) and is an essential component of adaptive immunity. B cells account for approximately 10% of total lymphocytes in the peripheral blood of healthy humans (1), and they consist of a repertoire of B cells expressing distinct BCRs to recognize diverse antigens, thereby playing important roles in effective humoral immune responses. The BCR is a heterotetrameric protein complex composed of two identical heavy chain proteins and two identical light chain proteins (2). The antigen-binding site of the heavy chain gene is composed of multiple distinct gene segments corresponding to the variable region (VH), diversity region (DH), and joining region (JH) (3). The variable regions of the light chains (κ and λ) are also composed of the variable segments (Vκ or Vλ) and the joining segments (Jκ or Jλ) (4). These gene segments generate BCR diversity through site-specific V(D)J recombination (5). During recombination, random deletions and non-templated insertions of nucleotides frequently occur at the junctions of each segment (6), markedly increasing BCR diversity. In this way, a unique BCR repertoire is formed for each individual.

Among the rearranged BCRs, those with autoreactivity are eliminated in the bone marrow, while those in the periphery encounter their cognate antigens and acquire further diversity through clonal affinity maturation (7). At this stage, helper T cell–mediated somatic hypermutation (SHM), induced by activation-induced cytidine deaminase, enhances antigen-binding affinity. Therefore, the BCR repertoire is known to reflect an individual’s immune status (6), and repertoire analysis is considered capable of providing next-generation biomarkers and diagnostic tools for various diseases, including autoimmune, infectious, and allergic diseases (8). For example, specific B cells within the repertoire are thought to differentiate into IgE-producing cells in patients with allergy (9), and the repertoire composition may change with the progression of allergy. Similarly, the repertoire composition is also thought to change as the disease progresses in other diseases (6).

On the other hand, because VDJ recombination and nucleotide insertion during B cell development have been considered to occur randomly, the relationship between the initial B cell repertoire and individual allergen specificity or susceptibility to a specific disease has not attracted much attention. However, it is known that the epitopes recognized by antibodies induced by the same antigen differ between strains of mice (10). Therefore, it cannot be ruled out that the initial B cell repertoire differs among strains or individuals and is associated with susceptibility to various diseases.

The objective of this study is to understand the differences in initial BCR repertoire among individuals and strains. Today, high-throughput immunoglobulin sequencing (IgSeq) technology is used for B cell repertoire analysis (4). This method enables the identification of the V(D)J segments used in each B cell, the analysis of SHM rates, and the characterization of the length and sequence of the complementarity-determining region 3 (CDR3), which is a particularly important region for antigen recognition (11). Although many studies have analyzed BCR repertoires in patients with specific diseases, including autoimmune diseases and allergies (6, 12, 13), most have focused on repertoires acquired through patients’ immune responses. Consequently, there are few studies targeting the BCR repertoire in healthy individuals (8, 14, 15). Therefore, it remains unclear whether the innate repertoire of healthy individuals differs according to genetic background. However, it is impossible to analyze the original BCR repertoire in large numbers of genetically identical individuals or in individuals raised under identical dietary and environmental conditions in human studies. Therefore, in this study, we analyzed the innate BCR repertoire across several mouse strains. A previous study reported that the frequency of IGHV gene usage in naive IgM-producing B cells obtained from murine spleens showed no major changes in distribution from 2 to 8 weeks of age (16). Based on this evidence, we analyzed IGHV and IGKV gene usage at the individual level using splenic IgM-expressing B cells from three mouse strains—BALB/c, C57BL/6N, and NC/Nga—to investigate inter-individual and inter-strain differences in the innate BCR repertoire under healthy conditions.

This approach will clarify whether the BCR repertoire in the healthy state is randomly generated or inherently biased across individuals based on genetic background. The findings of this study are expected to provide an important foundation for understanding the mechanisms underlying inter-individual differences in susceptibility to various immune-related diseases and resistance to diverse infections.

## 2 Materials and methods

### 2.1 Mice

BALB/c, C57BL/6N, and NC/Nga mice were purchased from Japan SLC (Shizuoka, Japan), three per strain. All individuals were 5 weeks old and female. Mice were euthanized by cervical dislocation immediately upon arrival and used in subsequent experiments. All procedures were conducted in accordance with the Tokyo University of Agriculture and Technology Guidelines for Laboratory Animal Care and Use (Approval Numbers: R05-20, R06-15, R07-12).

### 2.2 Cells

The spleen was aseptically removed from mice in a clean bench and thoroughly dissociated on a dish containing RPMI 1640 (Shimadzu Diagnostics, Tokyo, Japan). The cell suspension was filtered through a #200 nylon mesh. After centrifugation (4°C, 150×g, 5 min), the supernatant was discarded. RPMI medium was added, the cells were resuspended and then centrifuged again. The supernatant was removed again, and the cells were resuspended in RPMI medium supplemented with 10% fetal bovine serum (FBS, Sigma, Germany). Viable cells were counted with a microscope after trypan blue staining.

### 2.3 Flow cytometry

Murine splenocytes were stained at 4°C for 30–40 min with the following mouse surface antigen–specific monoclonal antibodies: anti-IgM-PE antibody (RMM-1, BioLegend, San Diego, USA) and anti-B220-PECy5 antibody (RA3-6B2, BioLegend). Each IgM-expressing B cell was sorted using a flow cytometer (FACSAria™ III, BD, NJ, USA) into a 96-well PCR plate containing 4 μL/well of ice-cold 0.5× phosphate-buffered saline (PBS) supplemented with 0.01 M DTT (Invitrogen, Carlsbad, CA, USA), 8 U RNaseOUT™ (Invitrogen). The FACS gating strategy used during cell isolation was shown in **Supplementary Figure 1**. The plates were sealed, immediately frozen, and stored at −80°C.

### 2.4 Single cell sequencing

cDNA was synthesized in the original 96-well sorting plate at a total volume of 14 µL per well, as previously described (17). In detail, total RNA from each single cell was reverse-transcribed in nuclease-free water using 150 ng random hexamer primers (pd(N)6, Takara, Japan), 0.5 µL of 10 mM each dNTP mix, 1 µL of 0.1 M DTT, 2.3 µL of 5× First-Strand (FS) buffer (Invitrogen), 8 U RNase OUT (Invitrogen), and 50 U SuperScript™ (TM) IV reverse transcriptase (Invitrogen). The reverse transcription (RT) reaction was performed at 25°C for 10 min, 42°C for 10 min, 25°C for 10 min, 50°C for 60 min, and 94°C for 5 min.

Transcripts of IGHV and IGKV genes were independently amplified by nested PCR initiated with 1 μL of the cDNA solution as a template. All PCR reactions were carried out in 96-well plates at a total volume of 9 μL per well, containing 100 nM of each primer or primer mix (**Table 1**), 0.2 mM of each dNTP (Invitrogen), and 0.25 U HotStarTaq^®^ (R) DNA polymerase (Qiagen, Hilden, Germany). All PCR reactions were performed using nuclease-free water. All nested PCR reactions using gene-specific primers or primer mixes were performed with 1 μL of unpurified first-round PCR product. Each round of PCR consisted of 50 cycles of 94°C for 30 s, 52°C for 30 s, and 72°C for 55 s (first PCR) or 45 s (second PCR).

**Table 1:** Primer sequences.

PCR step	Primer name	5’-3’ sequence
Igh 1st PCR	5’ MsVHE	GGGAATTCGAGGTGCAGCTGCAGGAGTCTGG
	3’ Cµ outer	AGGGGGCTCTCGCAGGAGACGAGG
Igh 2^nd^ PCR	5’ MsVHE	GGGAATTCGAGGTGCAGCTGCAGGAGTCTGG
	3’ Cµ inner	AGGGGGAAGACATTTGGGAAGGAC
Igκ 1^st^ PCR	5’ L-Vκ mix	TGCTGCTGCTCTGGGTTCCAG ATTWTCAGCTTCCTGCTAATC TTTTGCTTTTCTGGATTYCAG TCGTGTTKCTSTGGTTGTCTG ATGGAATCACAGRCYCWGGT TCTTGTTGCTCTGGTTYCCAG CAGTTCCTGGGGCTCTTGTTGTTC CTCACTAGCTCTTCTCCTC
	3’ mCκ	GATGGTGGGAAGATGGATACAGTT
Igκ 2^nd^ PCR	5’ mVkappa	GAYATTGTGMTSACMCARWCTMCA
	3’ m JK mix	GCCACCGTACGTTTGATTTCCAGCTTGGTG GCCACCGTACGTTTTATTTCCAGCTTGGTC GCCACCGTACGTTTTATTTCCAACTTTGTC GCCACCGTACGTTTCAGCTCCAGCTTGGTC

Aliquots of the second-round PCR products of VH and Vκ chains were purified using ExoSAP-IT™ (TM) (USB Corporation, Ohio, USA) according to the manufacturer’s instructions, and nucleotide sequences were determined using the respective reverse primers (Fasmac, Tokyo). Sequences were analyzed using IgBLAST against GenBank (http://www.ncbi.nlm.nih.gov/igblast/), and the germline V(D)J gene segments with the highest identity were identified.

### 2.5 Analysis of the BCR repertoire

We analyzed the BCR repertoire for each individual using the obtained sequences and compared them across strains. Detected segments were denoted like IGHV5-4*02, but since the numbers after the asterisk (*) differ between strains, we used the portion before the asterisk (e.g., IGHV5-4) for some analyses.

### 2.6 Diversity and similarity of the BCR repertoire

The diversity of each lineage segment was measured using Shannon’s diversity index. Shannon’s diversity index (H′) was calculated using the following formula.


$$H^{\prime }=-\sum_{i}P_{i}\log_{\text{e}}\left(P_{i}\right),\;P_{i}=\frac{n_{i}}{N}$$

Additionally, the similarity of each individual’s repertoire was calculated using Jaccard similarity, and a heatmap was created. This analysis was followed by t-SNE dimensionality reduction to visualize similarity between individuals (18). Classical multidimensional scaling (MDS) was also performed using the Jaccard distance matrix (1 - Jaccard similarity) to visualize the overall relationships among individuals while preserving pairwise distances as closely as possible (19). For similarity calculation, the portion of the obtained segments before the asterisk was used for analysis. The Jaccard similarity (J) was calculated using the following formula.


$$J\left(A,B\right)=\frac{\left|A\cap B\right|}{\left|A\cup B\right|}$$

Jaccard similarity values are normalized to a range of 0 to 1, where 1 indicates completely identical sets, and 0 indicates sets with no common elements. Furthermore, t-SNE and MDS plots show that greater distance corresponds to lower similarity values. t-SNE and MDS were used to intuitively illustrate the relationships between samples based on Jaccard similarity, not to statistically estimate clusters.

### 2.7 Visualization of pairing data for variable region segments

To visually analyze the pairing patterns of gene segments between IGHV and IGKV, a circular visualization method was employed.

Using Python (v3.10) and the Plotly library (plotly.graph_objects), we plotted the Vκ segments paired with specific IGHV segments (e.g., IGHV5-4) as a circular network. Here, we also used the segment regions before the asterisk for analysis. Each VH segment was placed in the upper half of the circle (colored portion), while Vκ segments were placed in the lower half (gray portion). Links were extracted from the segments, and the connections to corresponding Vκ segments were visualized for each individual. The intensity of the link color indicates the number of detected combinations; darker colors indicate more frequently detected combinations.

### 2.8 Quantification of biases in variable region segment pairing

Furthermore, to quantify the degree of bias in the paired Vκ segments for the top 30 of the most frequently detected VH segments, a constraint index was used. After excluding individuals with detection counts of 5 or fewer, the number of VH segments analyzed was reduced to 13. The index was calculated using Shannon’s diversity index, as described above, with the following formula.


$$\text{Constraint}\left(\text{IGHV}\right)=1-\frac{H\left(\text{IGKV }|\text{ IGHV}\right)}{H\left({\text{IGKV}}_{\text{all}}\right)}$$

- H: Shannon entropy.
- H(IGKV_all_): Diversity of IGKV across the entire population.
- H(IGKV | IGHV): Diversity of IGKV conditioned on a specific IGHV.

This metric corresponds to the uncertainty coefficient (Theil’s U) in information theory. Values closer to 0 indicate weaker constraints imposed by IGHV, indicating that it hardly influences IGKV selection. Values closer to 1 indicate stronger constraints, meaning pairing with a specific IGKV.

Additionally, for the top 6 most frequently detected VH segments, we calculated the detection frequency and similarity of the corresponding Vκ segments. Detection frequency was visualized as stacked bar graphs, and similarity was visualized as a heatmap using the Jaccard similarity coefficient described above.

### 2.9 Statistics

Statistical analysis was performed by the Tukey-Kramer test following the one-way analysis of variance (ANOVA). Differences were considered significant when the probability value (P) < 0.05.

## 3 Results

### 3.1 Analysis of IGHV and IGKV diversity and CDR3 length in IgM B cells obtained from naive mice of different individuals

VH- and Vκ-positive IgM-expressing B cells were obtained from each of the three individuals in the three different strains (BALB/c: 132, 142, and 129 cells/mouse; C57BL/6N: 132, 123, and 127 cells/mouse; NC/Nga: 90, 127, and 89 cells/mouse). The number of detected VH segment types was BALB/c: 63, 36, and 33 per mouse; C57BL/6N: 51, 47, and 52 per mouse; and NC/Nga: 49, 42, and 43 per mouse. The number of detected Vκ segment types was BALB/c: 47, 40, and 46 per mouse; C57BL/6N: 48, 48, and 43 per mouse; and NC/Nga: 38, 47, and 37 per mouse. Detection frequencies of VH (**Figure 1**) and Vκ (**Figure 2**) segments were calculated for each individual and displayed as pie charts.

**Figure 1:**
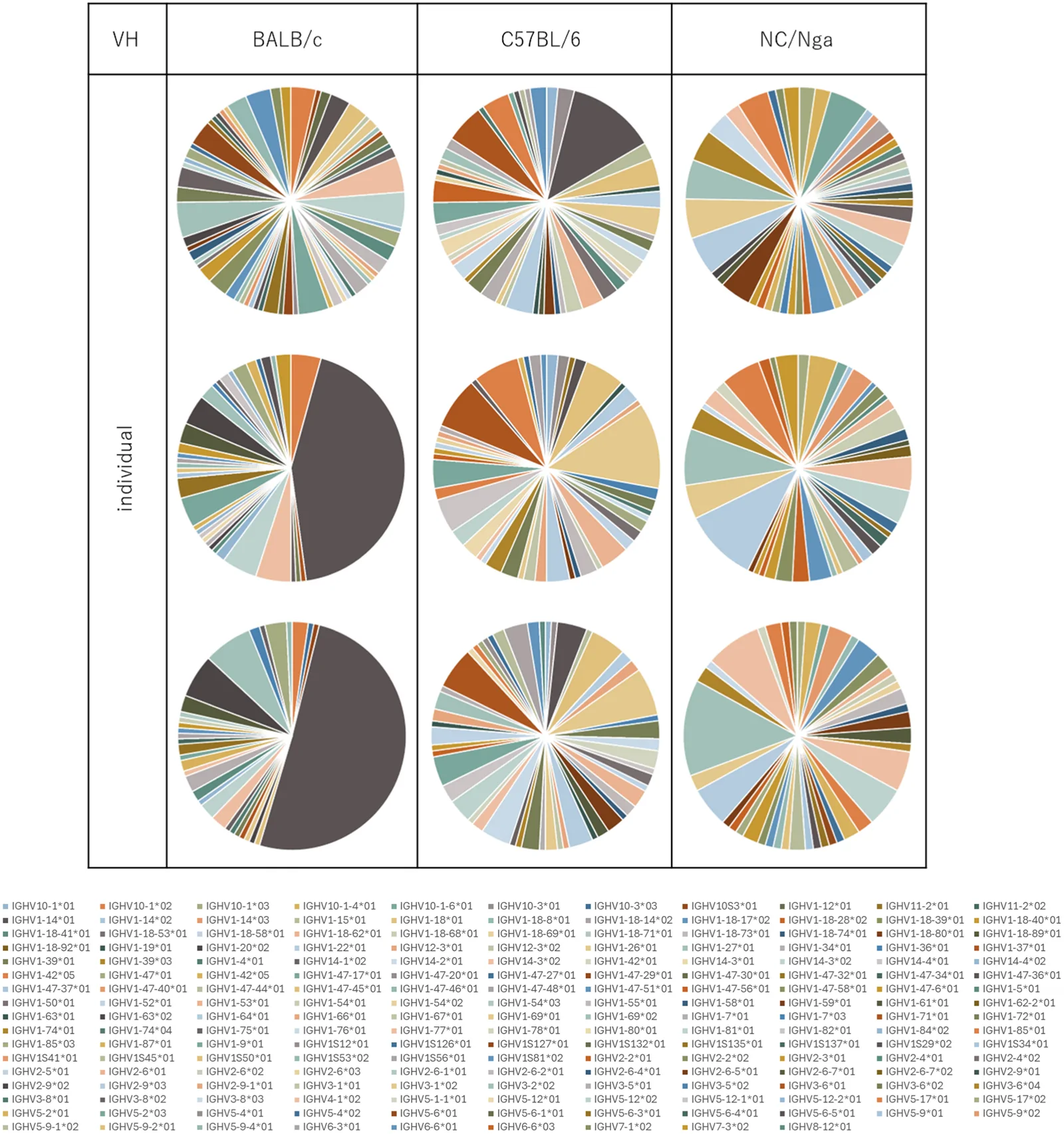
Composition of VH segments in the IgM BCR repertoire of each individual. The detection frequencies of IGHV segments were calculated for each individual and summarized in pie charts.

**Figure 2:**
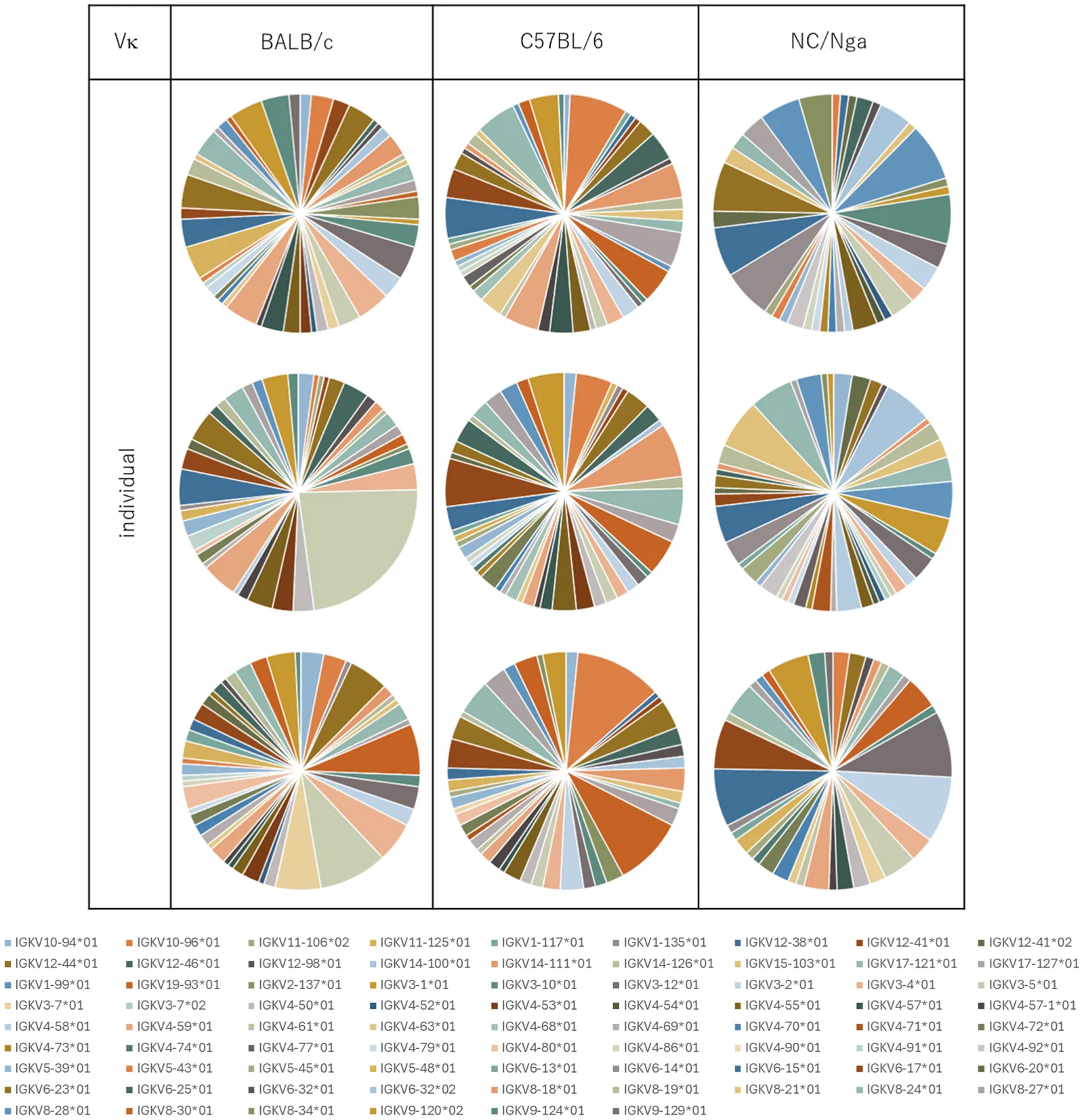
Composition of Vκ segments in the IgM BCR repertoire of each individual. The detection frequencies of IGKV segments were calculated for each individual and summarized in pie charts.

Next, the diversity of segments in each strain was evaluated. Significant difference in the diversity was observed among strains for neither VH nor Vκ segments (**Figure 3**). In **Figure 3A**, the diversity in BALB/c appeared slightly reduced; this was because a specific VH segment accounted for nearly half of the detected VH segments in two individuals, as demonstrated above (**Figure 1**).

**Figure 3:**
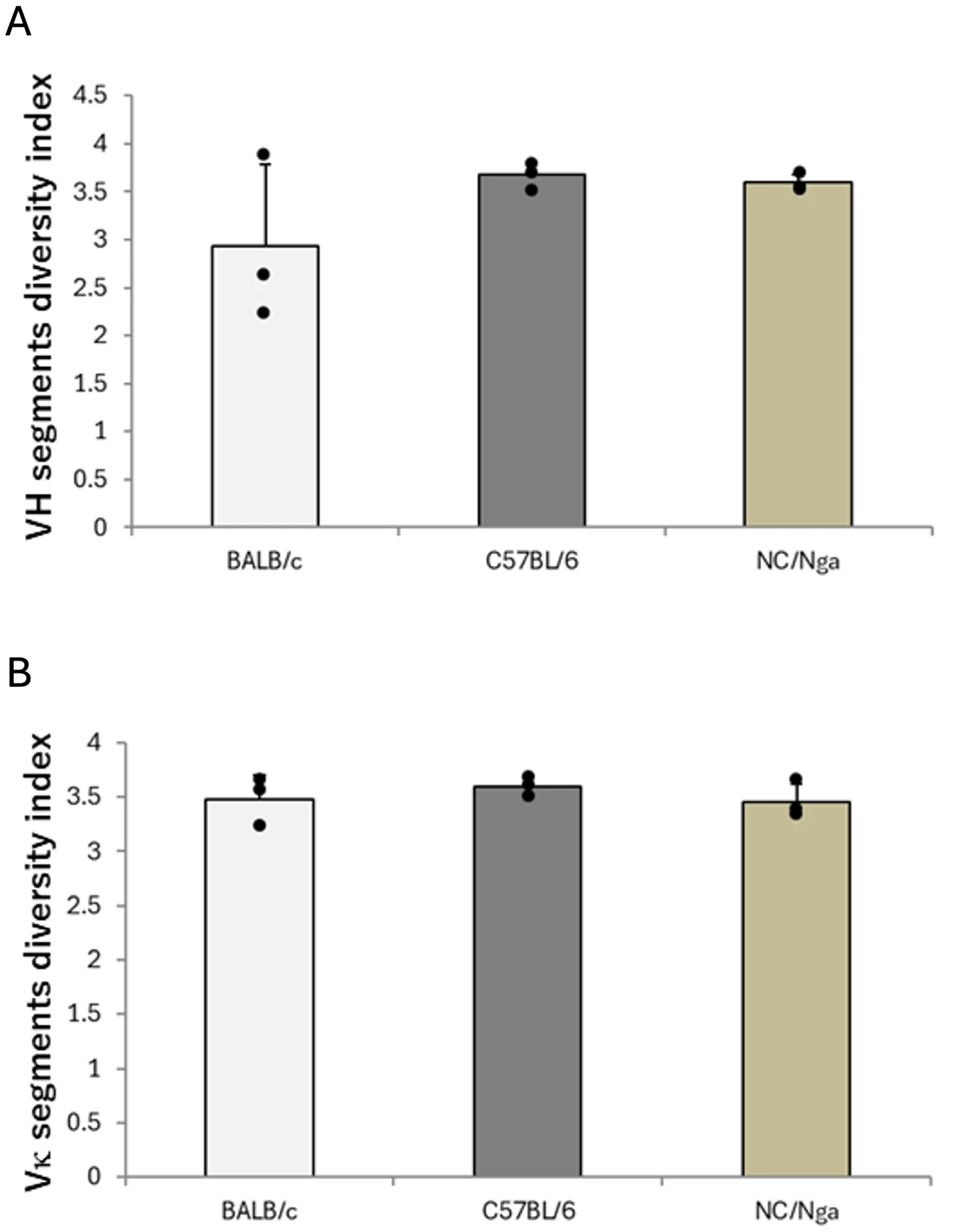
Segment diversity in the IgM BCR repertoire of each strain. **(A)** The diversity of VH segments was calculated for each individual using the Shannon diversity index. The results are presented as mean ± SD. **(B)** The diversity of Vκ segments was calculated for each individual using the Shannon diversity index. Results are presented as mean ± SD.

We then analyzed the average CDR3 length of each individual and compared it among strains. As a result, although two BALB/c mice showed shorter CDR3 lengths because the CDR3 length of the BCR in the abnormally highly detected VH segment was shorter than that of others, other individuals had lengths around 35 bp, with no differences among strains (**Figures 4A–D**). Similarly, for the average amino acid length of the region, two BALB/c mice showed shorter lengths, while other individuals were approximately 11 amino acids long, again with no differences among strains (**Figures 4E–H**). Furthermore, we examined whether any CDR3 amino acid sequences were shared among individuals or strains. A total of 329, 364, and 305 unique CDR3 amino acid sequences were identified in BALB/c, C57BL/6N, and NC/Nga mice, respectively. However, only one sequence was shared among all three strains, and even within the same strain, shared CDR3 amino acid sequences were rarely observed (**Figure 5**).

**Figure 4:**
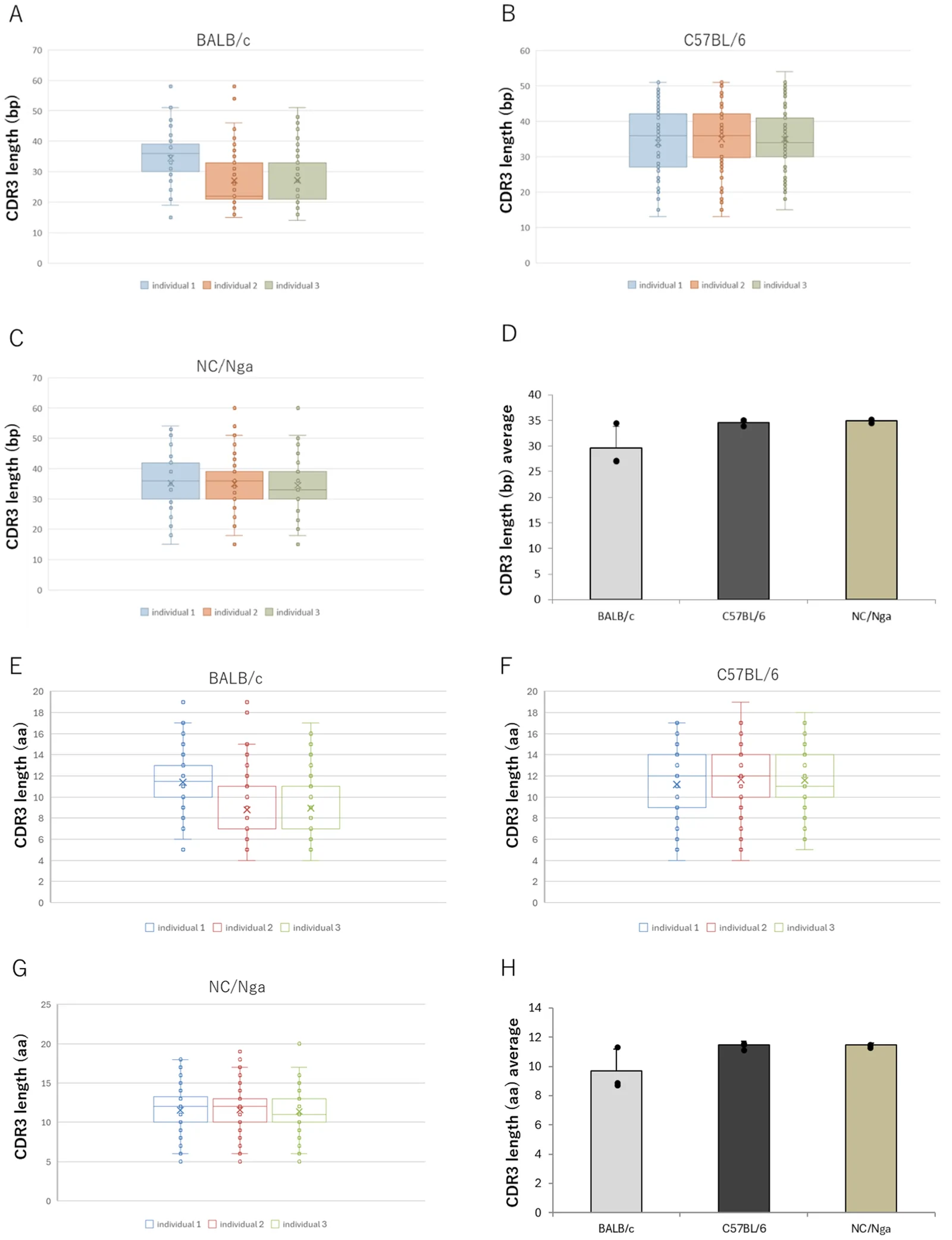
CDR3 length in the IgM BCR repertoire of each individual. Heavy-chain CDR3 length and amino acid length were compared among strains. **(A–C)** CDR3 nucleotide length (bp) was compared among individuals: **(A)** BALB/c, **(B)** C57BL/6N, and **(C)** NC/Nga. **(D)** To compare CDR3 nucleotide length (bp) among strains, results are presented as mean ± SD, and statistical significance between groups is indicated at p < 0.05. **(E–G)** Amino acid length was compared among individuals: **(E)** BALB/c, **(F)** C57BL/6N, and **(G)** NC/Nga. **(H)** To compare amino acid length among strains, results are presented as mean ± SD, and statistical significance between groups is indicated at p < 0.05.

**Figure 5:**
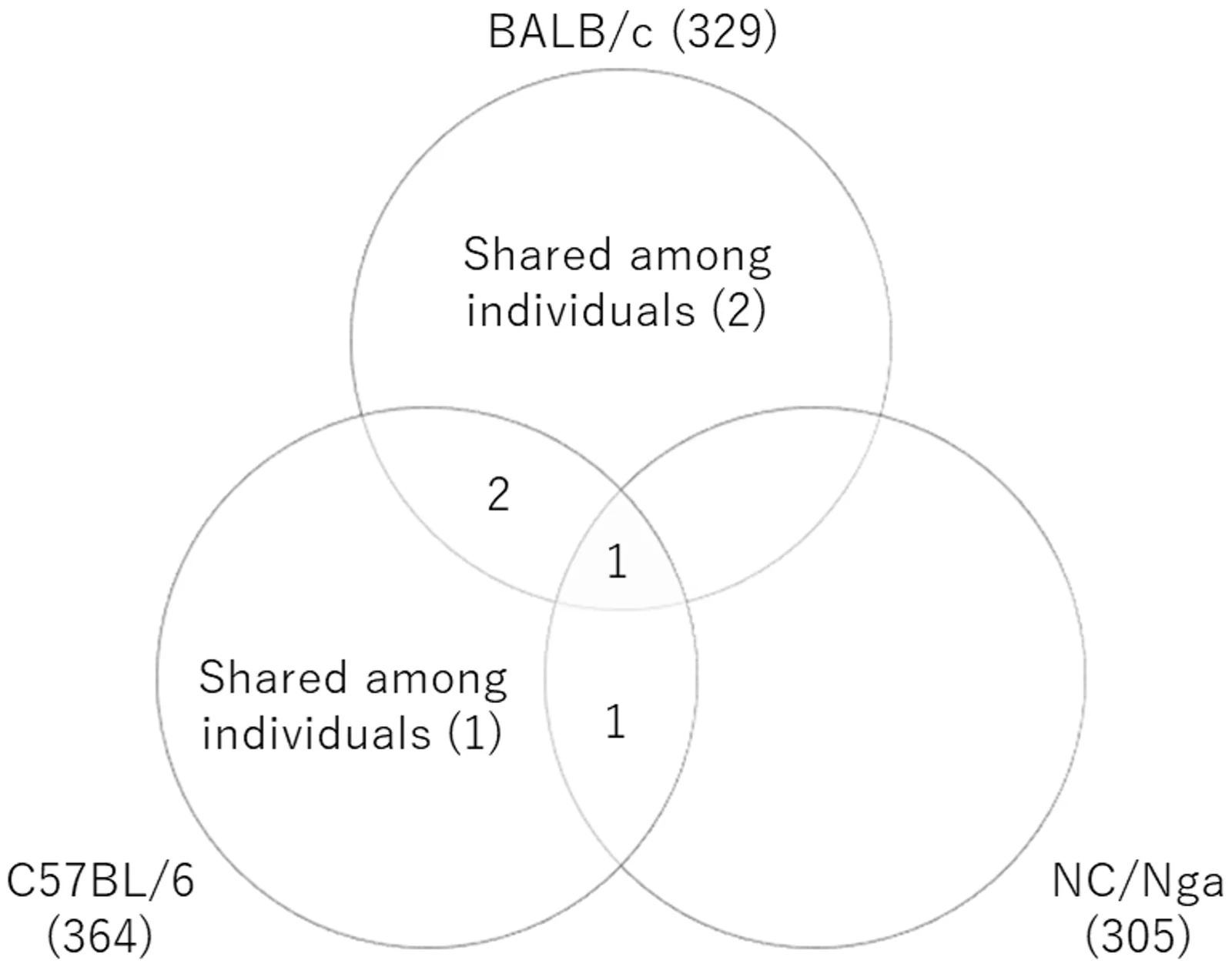
Shared CDR3 amino acid sequences in the IgM BCR repertoire of each strain. The number of shared heavy-chain CDR3 amino acid sequences among individuals and strains was calculated based on sequences detected in each individual.

### 3.2 Analysis of IGHV and IGKV similarity among strains

For the region before the asterisk in the VH segment names, IGHV genes from the IgM BCR repertoires of all individuals were listed. Multiple VH segments common to all three individuals in the strain but not observed in other strains were identified (**Table 2**). On the other hand, for IGKV genes, no Vκ segment common to all three individuals in the strain but absent in other strains was found, and most were shared among strains (**Table 3**).

**Table 2:** IGHV obtained from each individual (shaded segments indicate those detected in all three mice of one strain but not in other strains.).

	BALB/c			C57BL/6			NC/Nga		
	1	2	3	1	2	3	1	2	3
IGHV1-12	○								
IGHV1-14	○	○	○	○	○	○	○	○	○
IGHV1-15				○		○			
IGHV1-18	○		○	○	○	○	○		○
IGHV1-18-14							○		
IGHV1-18-17								○	○
IGHV1-18-28							○		
IGHV1-18-39								○	○
IGHV1-18-40							○		
IGHV1-18-41							○	○	
IGHV1-18-53							○		
IGHV1-18-58							○		
IGHV1-18-60								○	
IGHV1-18-62								○	○
IGHV1-18-68							○	○	○
IGHV1-18-69									○
IGHV1-18-71							○		
IGHV1-18-73							○		○
IGHV1-18-74							○	○	○
IGHV1-18-8									○
IGHV1-18-80									○
IGHV1-18-89							○	○	○
IGHV1-18-92								○	
IGHV1-19				○	○				
IGHV1-20			○					○	
IGHV1-22				○	○	○			
IGHV1-26	○		○	○	○	○			
IGHV1-27	○								
IGHV1-34				○					
IGHV1-36					○	○			
IGHV1-37	○	○	○						
IGHV1-39	○	○	○	○	○	○	○		○
IGHV1-4	○	○	○		○				
IGHV1-42				○		○			○
IGHV1-47	○				○				
IGHV1-47-17							○		○
IGHV1-47-20								○	○
IGHV1-47-27							○	○	○
IGHV1-47-29									○
IGHV1-47-30								○	○
IGHV1-47-32							○	○	○
IGHV1-47-34							○	○	
IGHV1-47-36							○	○	○
IGHV1-47-37							○	○	○
IGHV1-47-40							○	○	
IGHV1-47-44							○	○	○
IGHV1-47-45							○	○	○
IGHV1-47-46								○	○
IGHV1-47-48							○		
IGHV1-47-51							○	○	○
IGHV1-47-56							○	○	
IGHV1-47-58							○	○	○
IGHV1-47-6							○	○	○
IGHV1-5	○	○	○	○					
IGHV1-50				○	○	○			
IGHV1-52					○	○			
IGHV1-53				○	○	○			
IGHV1-54		○		○	○				
IGHV1-55	○			○	○	○			
IGHV1-56						○			
IGHV1-58				○	○	○			
IGHV1-59				○	○	○			
IGHV1-61				○		○			
IGHV1-62-2		○				○			
IGHV1-63		○		○		○			
IGHV1-64				○	○	○			
IGHV1-66	○				○	○			
IGHV1-67		○		○	○	○			
IGHV1-69	○			○	○	○			
IGHV1-7	○	○	○	○		○	○		
IGHV1-72				○	○	○			
IGHV1-74	○		○	○	○	○			
IGHV1-75						○			
IGHV1-76	○			○	○	○			
IGHV1-77			○	○	○	○			
IGHV1-78				○		○			
IGHV1-80	○	○		○	○				
IGHV1-81				○	○	○			
IGHV1-82	○	○		○	○	○			
IGHV1-84		○							
IGHV1-85		○	○		○		○		○
IGHV1-87	○	○	○				○		
IGHV1-9	○	○	○	○	○	○			
IGHV10-1	○	○	○	○	○	○	○	○	○
IGHV10-1-4							○	○	○
IGHV10-1-6							○	○	○
IGHV10-3			○	○	○	○			
IGHV10S3	○		○						
IGHV11-2					○				
IGHV12-3	○				○	○			
IGHV14-1	○	○	○				○		
IGHV14-2				○	○	○			
IGHV14-3	○	○	○	○			○	○	○
IGHV14-4	○	○	○	○		○			
IGHV1S12	○								
IGHV1S127	○								
IGHV1S132	○								
IGHV1S135	○	○	○						
IGHV1S137	○		○						
IGHV1S29	○								
IGHV1S34	○	○							
IGHV1S41	○								
IGHV1S45	○								
IGHV1S50		○							
IGHV1S53	○	○							
IGHV1S56		○	○						
IGHV1S81	○	○	○						
IGHV2-2	○			○	○	○	○	○	○
IGHV2-3	○	○	○		○	○	○	○	
IGHV2-4	○			○					
IGHV2-5					○	○			
IGHV2-6		○	○	○	○	○			
IGHV2-6-1	○		○						
IGHV2-6-4	○								
IGHV2-6-5	○						○	○	○
IGHV2-6-7		○	○				○		
IGHV2-9	○	○	○	○		○	○	○	○
IGHV2-9-1				○	○	○			
IGHV3-1				○			○	○	○
IGHV3-2	○	○	○	○		○	○	○	○
IGHV3-5		○	○	○	○	○			
IGHV3-6	○			○	○	○	○	○	○
IGHV3-8	○	○	○	○	○		○	○	○
IGHV4-1							○	○	○
IGHV5-1-1								○	○
IGHV5-12	○					○			
IGHV5-12-1		○							
IGHV5-12-2	○	○							
IGHV5-15								○	
IGHV5-17	○	○	○	○	○	○	○	○	○
IGHV5-2		○		○	○				
IGHV5-4	○	○			○	○	○		○
IGHV5-6	○	○		○	○				
IGHV5-6-3	○								
IGHV5-6-4	○								
IGHV5-6-5	○	○		○					
IGHV5-9	○					○			
IGHV5-9-1				○		○			
IGHV5-9-2	○								
IGHV5-9-4	○	○	○						
IGHV6-3				○	○	○		○	
IGHV6-6	○			○	○	○		○	○
IGHV7-1	○			○			○	○	○
IGHV7-3	○	○		○		○	○	○	
IGHV8-12						○			
IGHV8-4-8								○	

**Table 3:** IGKV obtained from each individual.

	BALB/c			C57BL/6			NC/Nga		
	1	2	3	1	2	3	1	2	3
IGKV1-110								○	
IGKV1-117				○					
IGKV1-135			○		○				
IGKV1-99				○					
IGKV10-94	○	○	○	○	○	○	○	○	
IGKV10-96	○	○	○	○	○	○		○	○
IGKV11-106		○					○		
IGKV11-125					○				
IGKV12-38				○		○			
IGKV12-41	○	○		○	○	○	○		
IGKV12-44	○	○	○	○	○	○	○	○	○
IGKV12-46	○	○		○	○	○		○	
IGKV12-98	○	○		○		○	○		○
IGKV14-100	○				○	○	○	○	
IGKV14-111	○	○	○	○	○	○	○	○	○
IGKV14-126	○	○	○	○	○			○	○
IGKV15-103	○		○	○		○		○	
IGKV17-121	○	○	○	○	○	○	○	○	○
IGKV17-127	○	○	○	○	○	○		○	○
IGKV19-93	○	○	○	○	○	○	○	○	○
IGKV2-137	○	○				○			
IGKV3-1	○						○		
IGKV3-10	○	○	○	○	○	○	○	○	○
IGKV3-12	○		○	○	○	○	○	○	○
IGKV3-2	○		○	○	○	○	○	○	○
IGKV3-3								○	
IGKV3-4	○	○	○	○	○	○	○	○	○
IGKV3-5	○	○	○	○	○	○	○	○	○
IGKV3-7	○		○		○	○	○	○	○
IGKV4-50	○	○	○	○	○	○		○	○
IGKV4-52	○		○						
IGKV4-53	○	○	○		○		○	○	
IGKV4-55	○	○	○	○	○	○	○	○	
IGKV4-57	○		○	○	○	○	○	○	○
IGKV4-57-1	○	○	○	○	○	○			○
IGKV4-58		○							
IGKV4-59	○	○	○	○	○	○	○	○	○
IGKV4-61				○		○			○
IGKV4-63	○		○	○	○				○
IGKV4-68				○	○				
IGKV4-69		○	○		○	○			
IGKV4-70	○		○		○		○	○	○
IGKV4-71						○	○		
IGKV4-72	○	○	○	○	○	○		○	○
IGKV4-73					○				
IGKV4-74					○		○	○	○
IGKV4-77				○					
IGKV4-79	○		○		○			○	
IGKV4-80		○	○			○	○	○	
IGKV4-86			○	○	○			○	
IGKV4-90						○	○	○	
IGKV4-91	○	○	○	○					
IGKV5-39		○	○	○	○	○	○	○	
IGKV5-43	○		○	○			○	○	
IGKV5-45				○	○	○	○		○
IGKV5-48	○	○	○		○	○	○	○	○
IGKV6-13			○	○	○				○
IGKV6-14		○						○	○
IGKV6-15	○	○	○	○	○	○	○	○	○
IGKV6-17	○	○	○	○	○	○	○	○	○
IGKV6-20		○	○		○			○	
IGKV6-23	○	○	○	○	○	○	○	○	
IGKV6-25		○	○		○		○	○	
IGKV6-32			○	○	○			○	
IGKV8-18				○					
IGKV8-19	○	○	○	○	○	○		○	○
IGKV8-21	○			○				○	
IGKV8-24	○	○	○	○	○	○	○	○	○
IGKV8-27	○	○			○	○	○	○	○
IGKV8-28	○	○		○	○	○	○	○	○
IGKV8-30	○		○	○	○	○	○	○	○
IGKV8-34						○			
IGKV9-120	○	○	○	○	○	○	○	○	○
IGKV9-123								○	
IGKV9-124	○	○	○	○				○	○
IGKV9-129	○								○

Therefore, repertoire similarity among individuals was calculated based on the segment name before the asterisk and visualized. For VH segments, similarity among individuals within the same strain was high (≥0.38, average 0.51). In contrast, similarity between different strains was <0.32 (**Figure 6A**). In t-SNE analysis, the characteristics of VH segments from the same strain were plotted in proximity. In contrast, those from different strains were more widely separated (**Figure 6B**). Similar results were obtained by using MDS analysis (**Figure 6C**). Statistical analysis of similarities among individuals within the same strain and between two different strains showed that inter-strain similarity was significantly lower than intra-strain similarity (**Figure 6D**).

**Figure 6:**
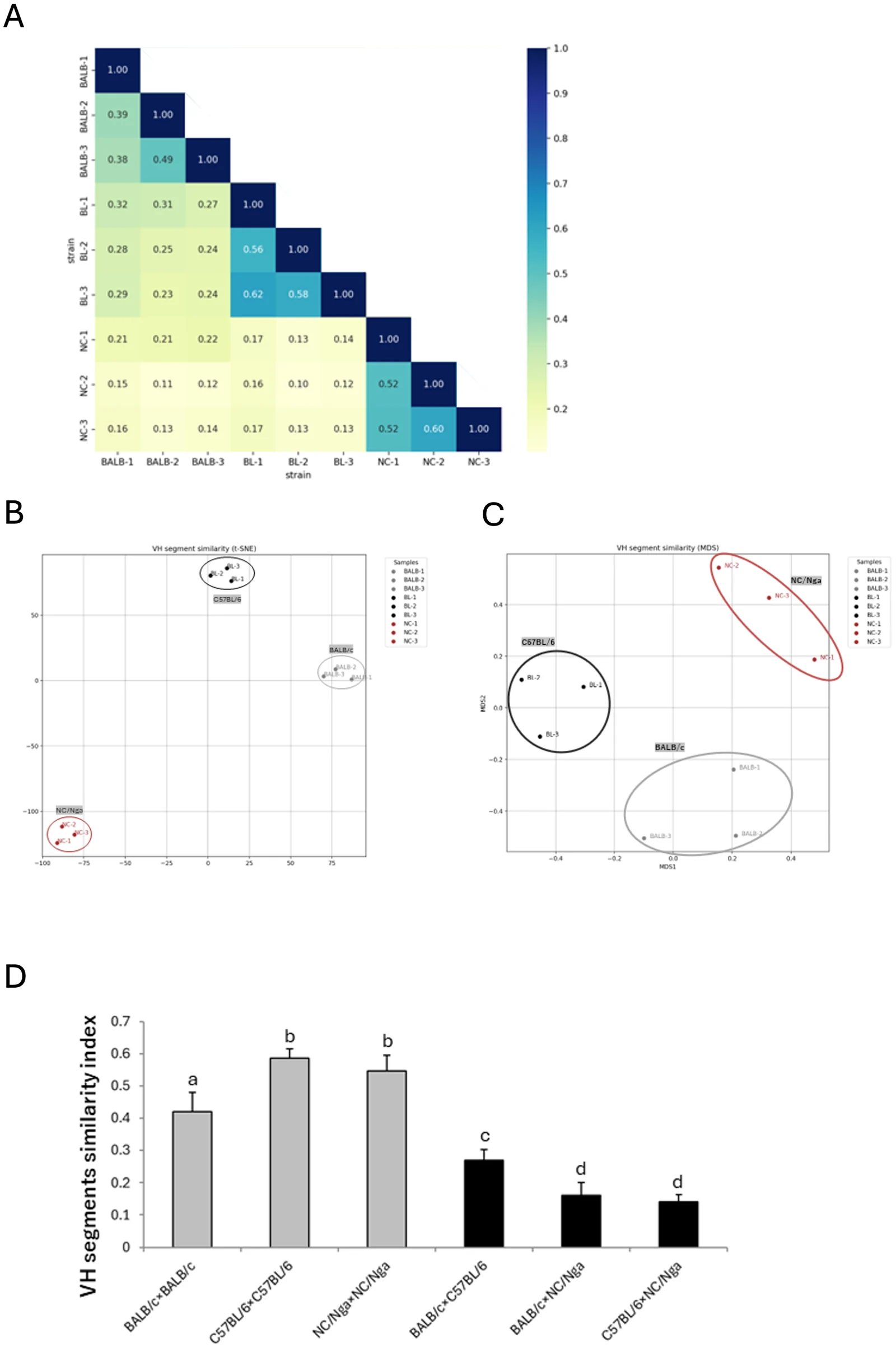
Similarity of VH segments in the IgM BCR repertoire of each individual. **(A)** Pairwise similarity between individuals was calculated using the Jaccard index. Values range from 0 to 1, with higher values indicating greater similarity. **(B, C)** To visualize similarity, distances between samples were calculated using the Jaccard index, and dimensionality reduction was performed using t-SNE **(B)** and MDS **(C)**. Shorter distances between plots indicate higher similarity. **(D)** A bar graph was generated from the Jaccard similarity values calculated in **(A)** to evaluate similarity within and between strains. Results are presented as mean ± SD, and statistical significance between groups is indicated at p < 0.05. Different letters indicate statistical significance.

In contrast, for Vκ segments, similarity values were ≥0.41 among all individuals, indicating only minor individual bias regardless of strain (**Figure 7A**). Moreover, in t-SNE and MDS analyses, the plots were dispersed by individual rather than by strain (**Figures 7B, C**), and no clear differences among strains were observed (**Figure 7D**).

**Figure 7:**
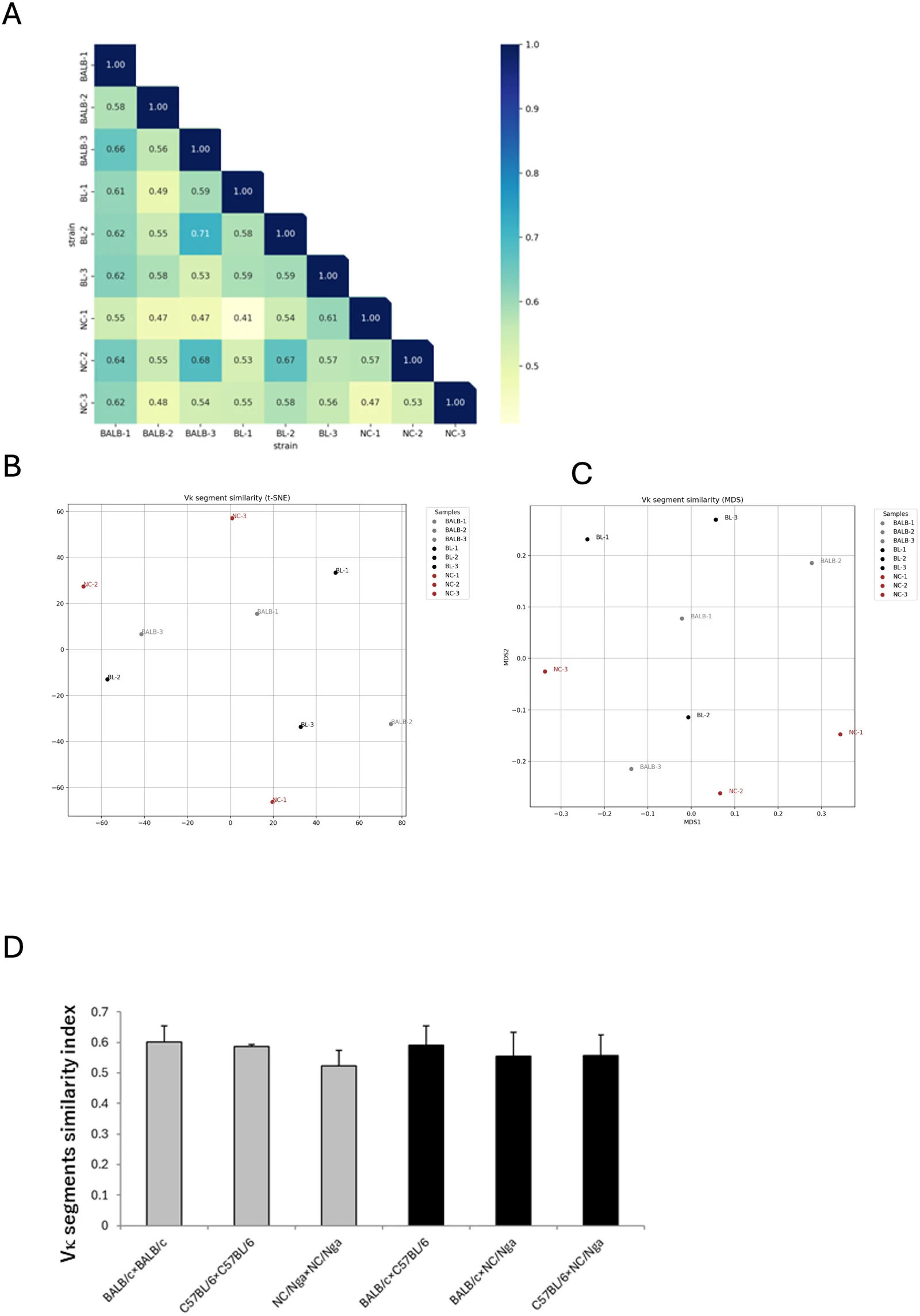
Similarity of Vκ segments in the IgM BCR repertoire of each individual. **(A)** Pairwise similarity between individuals was calculated using the Jaccard index. Values range from 0 to 1, with higher values indicating greater similarity. **(B, C)** To visualize similarity, distances between samples were calculated using the Jaccard index and dimensionality reduction was performed using t-SNE **(B)** and MDS **(C)**. Shorter distances between plots indicate higher similarity. **(D)** A bar graph was generated from the Jaccard similarity values calculated in **(A)** to evaluate similarity within and between strains. Results are presented as mean ± SD, and statistical significance between groups is indicated at p < 0.05.

### 3.3 Analysis of IGHV–IGKV pairing in the IgM BCR repertoire

Next, IGHV–IGKV pairing was analyzed using circular visualization. Strain-specific network patterns were observed, and the figure clearly showed that the combinations differed markedly among strains (**Figure 8**). Therefore, we quantified whether there was a bias in the Vκ segments selected by each VH segment (before the asterisk) using a constraint index for each individual. As a result, the average (± SD) constraint index was 0.45 ± 0.13, suggesting a potential bias in Vκ segment selection based on the VH segment (**Figure 9**).

**Figure 8:**
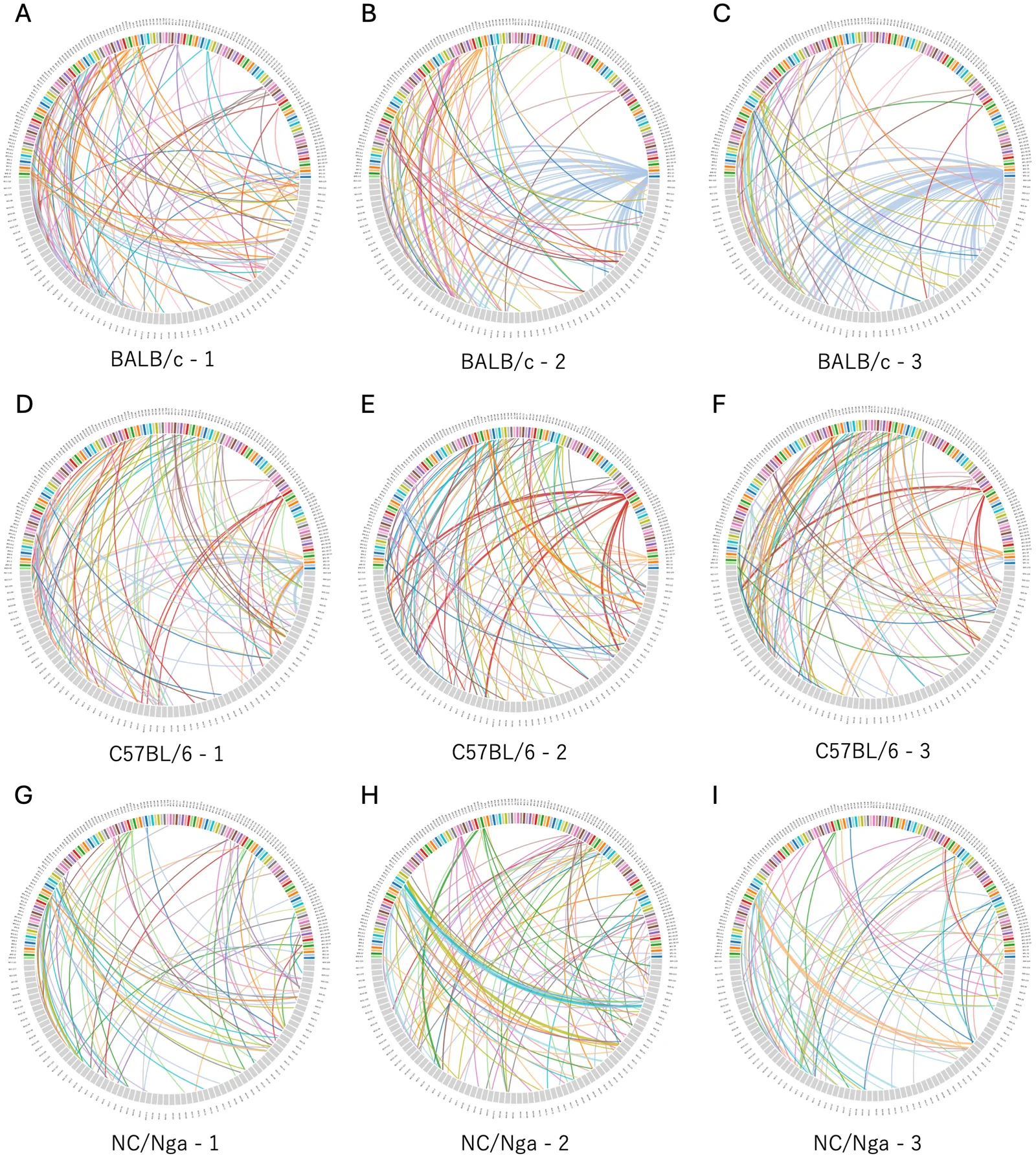
Circular network diagrams of combinations between IGHV and IGKV segments. To visualize IGHV–IGKV pairing, circular diagrams were generated for each individual. **(A–C)** BALB/c, **(D–F)** C57BL/6N, **(G–I)** NC/Nga.

**Figure 9:**
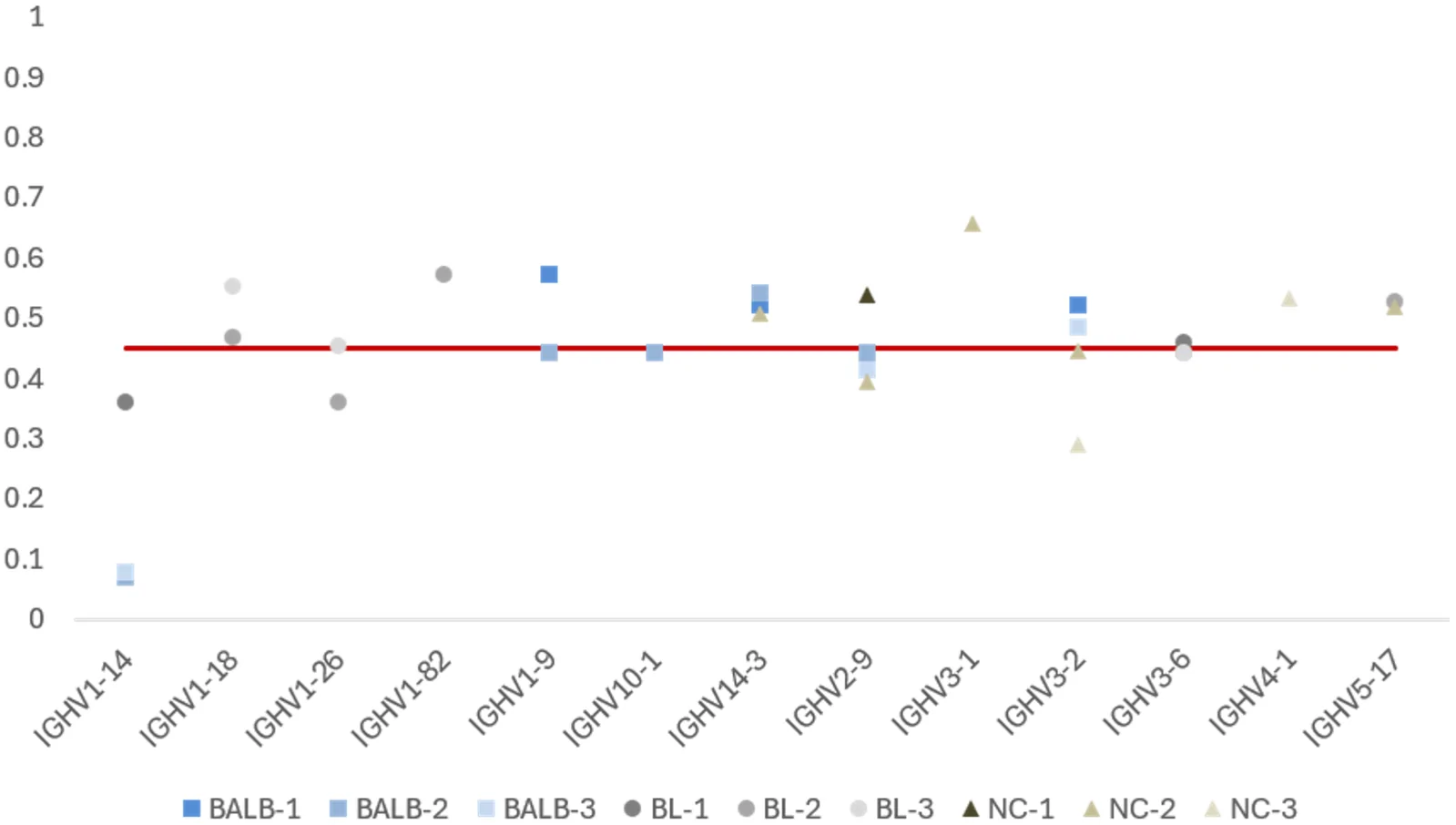
Constraint index of the Vκ segment in a specific VH segment. For the top 30 in most frequently detected VH segments, the degree of bias in paired Vκ segments were quantified using a constraint index. After excluding individuals with detection counts of 5 or fewer, the number of VH segments analyzed was reduced to 13. Values range from 0 to 1, with values closer to 1 indicating a stronger constraint, i.e., preferential pairing with specific IGKV segments. The red horizontal line indicate the overall mean constraint index (0.45).

Furthermore, we examined in detail, for the top six of the most frequently detected IGHV segments, whether the paired Vκ segments showed strain-dependent bias (**Figure 10**). For some VH segments, the selected Vκ segments showed high similarity within the same strain but lower similarity between strains (**Figures 10C, D**). Notably, IGHV2-9 showed significant differences in paired Vκ(symbol) segment selection between BALB/c and NC/Nga mice and between C57BL/6N and NC/Nga mice (**Figure 10C**). These results suggest that the paired Vκ segments against a specific VH segment might exhibit strain-dependent bias.

**Figure 10:**
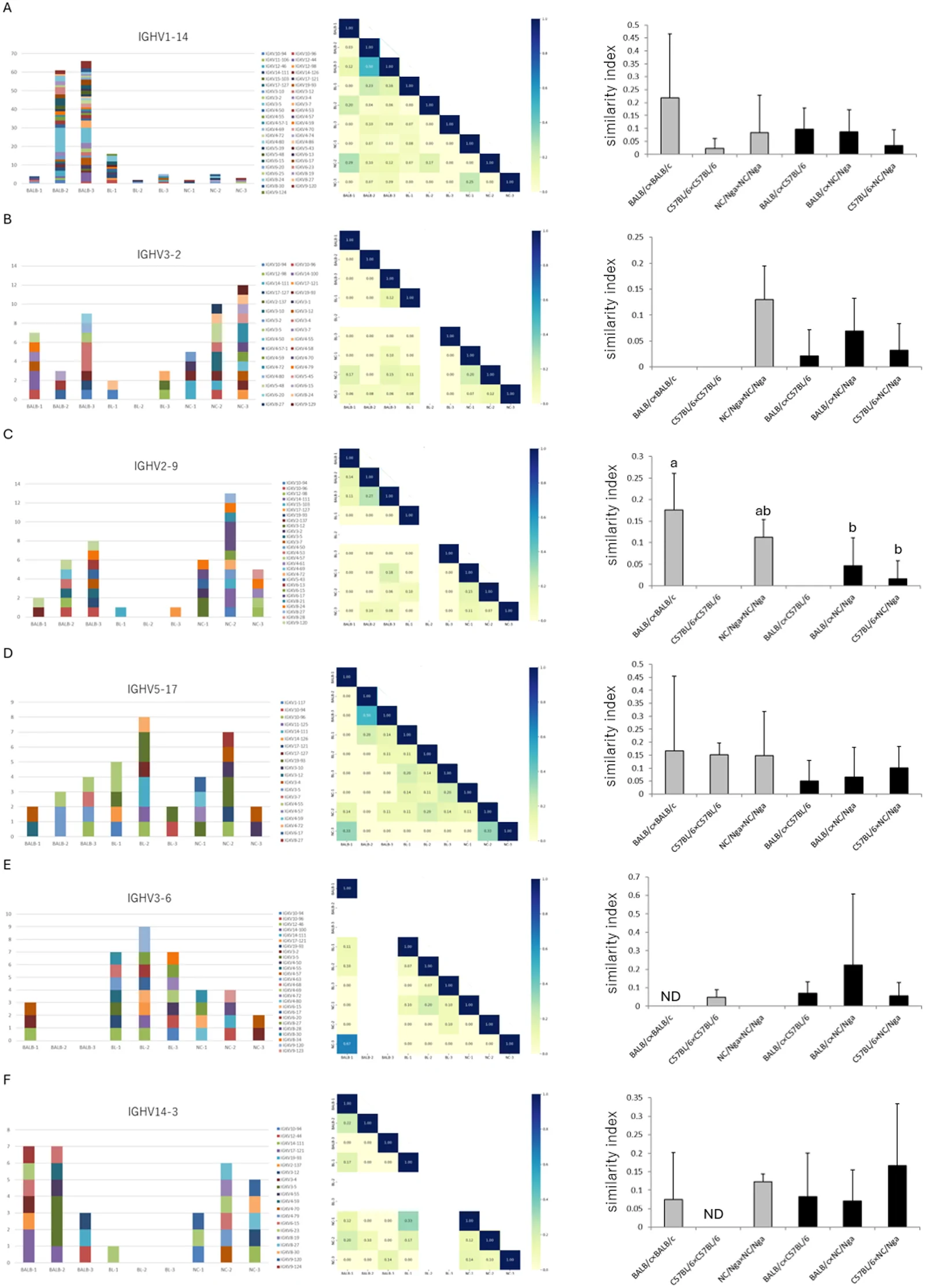
Strain difference in selectivity of specific Vκ segments by a VH segment. For the top six most frequently detected VH segments, the number of paired Vκ segments and their similarity among individuals and strains were analyzed. The number of paired Vκ segments was shown as stacked bar graphs, and similarity was visualized as a heatmap based on the Jaccard index, as in **Figure 6**. In addition, bar graphs were generated from the calculated Jaccard index values to evaluate similarity within and between strains. Results are presented as mean ± SD, and statistical significance between groups is indicated at p < 0.05. **(A)** IGHV1-14, **(B)** IGHV3-2, **(C)** IGHV2-9, **(D)** IGHV5-17, **(E)** IGHV3-6, **(F)** IGHV14-3.

## 4 Discussion

Although it is known that the murine BCR repertoire in naïve mice exhibits some inter-individual variation, the extent of variation between individuals and among strains has not been thoroughly investigated. Therefore, in this study, we examined differences in the IgM BCR repertoire in naïve mice using two inbred strains, BALB/c and C57BL/6N, as well as NC/Nga, a strain known to produce IgE antibodies readily. As a result, we demonstrated that the repertoire differed among strains but not among individuals.

In this study, a specific VH segment was detected at high frequency in two BALB/c individuals. Therefore, we recalculated the similarity of VH segments after excluding the segment from the repertoire data, but the change was small (**Supplementary Figure 2**). These findings suggest that the repertoire similarity within the same strain and the diversity among strains observed in this study did not result from the abnormal use of a single segment but rather reflected overall differences in repertoire composition. However, the reason why one specific VH segment was highly detected in these individuals remains unclear, and further investigation is needed. Incidentally, the CDR3 sequences of the abnormally highly expressed VH segment were identical in most cells from each individual, indicating that a single clone abnormally proliferated for some reason in the naïve mice. Although these mice were maintained under conventional conditions and showed no evidence of intentional antigen stimulation, spontaneous homeostatic clonal expansion or exposure to environmental antigens cannot be excluded. In any case, it is unlikely to have influenced the main conclusions of this study because exclusion of this expanded clone had little effect on repertoire similarity.

A particularly interesting finding was that the strain-dependent bias in V-segment composition was observed only in the heavy chain, not in the light chain. This trend is generally consistent with previous reports showing that the detection frequency of each heavy-chain V segment in naïve mice differed among strains. In contrast, those of light-chain V segments showed little inter-strain variation (20). These results indicate that genetic factors contributing to strain-dependent V gene selection play a major role in shaping each individual’s BCR repertoire.

Two possible explanations could account for the strain-dependent differences in VH segment selection observed in this study. The first concerns selection pressure during recombination. Kenter et al. demonstrated that the three-dimensional chromatin configuration and loop formation at the IGH locus differ among strains, leading to differences in RAG accessibility to each segment (21). The second concerns selection pressure during B cell development. Kaplinsky et al. showed that the VH repertoire was extensively reshaped by strong negative selection, which eliminates self-reactive clones during the transition from immature to mature B cells (22). The selectivity and strength (threshold) of this selection might differ among strains. Braun et al. further demonstrated that, even in knock-in mice carrying the same self-reactive BCR, the proportion of immature B cells eliminated by negative selection varied widely across genetic backgrounds (23). In other words, the extent of negative selection would be strain-dependent and reflect differences in the selecting environment rather than the BCR sequence itself. Taken together, the strain differences in VH segment usage observed in this study are likely generated through a combination of differences in recombinase accessibility and selection pressures at multiple developmental stages.

We also compared heavy-chain CDR3 nucleotide and amino acid lengths across strains and found that CDR3 length was similar across all strains. The average amino acid length in each individual was approximately 11, consistent with the report by Rettig et al. (24). Furthermore, analysis of shared amino acid sequences revealed only one sequence shared among the three strains, and 0–2 shared sequences even among individuals of the same strain. This finding is consistent with previous findings in C57BL/6J mice showing very few shared heavy-chain CDR3 sequences among individuals (24), indicating high inter-individual variability in CDR3 sequences that contribute to a high diversity of antigen specificity. In general, the heavy-chain CDR3 region directly contacts the antigen, and its length and sequence are thought to reflect structural diversity in antigen-binding modes and binding sites (16, 25). The present results suggest that heavy-chain CDR3 amino acid sequences are not the primary cause of strain-specific antigen recognition. Indeed, analysis of rodent antibody repertoires has shown that antibodies sharing the same VH/Vκ gene segment pairing recognized nearly identical epitopes even when their CDR3 sequences differed (26). Thus, small differences in the CDR3 amino acid sequence would not substantially alter epitope specificity and might mainly affect epitope affinity, suggesting that epitope specificity largely depends on VH/Vκ pairing.

Furthermore, regarding heavy–light chain pairing, this study suggested that different Vκ segments were preferentially selected depending on the strain, even when the same VH segment was used. This finding indicates that VH/Vκ pairing is not completely random and that genetically preferred combinations would exist. Jayaram et al. reported that VH/Vλ pairing shows a statistically significant bias and that certain combinations are structurally more stable (27). The strain differences observed in this study might indicate that such structural compatibility between heavy and light chains differed among strains. In other words, the most stable Vκ partner for a given VH segment might vary by strain, resulting in its own BCR repertoire formation. One possible explanation for structural compatibility is that the gene sequence for the same V segment slightly differs among strains because of allelic variants (indicated by the numbers after the asterisk of each segment name). Such bias in VH/Vκ pairing might contribute to the strain-specific differences in antigen recognition. However, because the overall usage frequency of Vκ did not differ among strains, the antigen specificity of each BCR is likely to depend primarily on VH segment selection. Nevertheless, reports on IGHV–IGKV pairing and its role remain limited. Another limitation of the present study is that only Igκ-expressing B cells were analyzed. Igλ-expressing B cells may exhibit distinct VH–Vλ pairing characteristics because λ-chain rearrangement occurs later during B-cell development and Igλ-expressing cells may include B cells that have undergone receptor editing. Future studies including Igλ-expressing B cells will provide a more comprehensive understanding of strain-dependent BCR repertoire formation. Nevertheless, in mice, approximately 95% of mature B cells express Igκ light chains, whereas only a small fraction express Igλ. Therefore, analysis of VH–Vκ pairing captured the major features of the murine naïve B-cell repertoire.

We understand further limitation of the present study is the relatively small sample size, both in terms of the number of mice analyzed and the number of paired BCR sequences obtained from each individual. Although consistent strain-dependent trends were observed, the present dataset may not fully capture the complete spectrum of VH–Vκ pairing patterns. Therefore, larger studies including more animals and a greater number of paired BCR sequences will be required to validate and extend the findings.

It has been reported that the onset of immune-mediated diseases was accompanied by changes in the B-cell repertoire, such as increased expression of specific VH segments (12). Rubelt et al. demonstrated that the proportions of many IGHV segments changed markedly after immune activation, providing strong evidence indicating that VH segment selection could directly influence antigen specificity (28). In addition, bias in VH segment composition has been reported to affect susceptibility to infectious diseases, B-cell lymphoma, and autoimmune diseases (29–31). Furthermore, studies using Ro-derived peptides of the Ro (SSA) protein—an autoantigen frequently detected in the sera of patients with systemic lupus erythematosus and Sjögren’s syndrome—have shown that epitope spreading differs among mouse strains even when the same immunogen was used (32). These findings support the possibility that diversification of antibody responses and the spread of epitope recognition would be strongly influenced by genetic background, especially VH selection. In other words, the results of this study strongly suggest that one reason antibody production against specific antigens differs among strains is the inherent differences in the pre-existing B-cell repertoire resulting from VH selection bias. The findings of this study are expected to provide important insights into how genetic background influences various immune-related diseases.

## Data Availability

The nucleotide sequences of the V regions obtained in this study have been submitted to DNA Data Bank of Japan (DDBJ) through the Mass Submission System (MSS); accession numbers LC948761–LC950942. Other data supporting the findings of this study are available from the corresponding author upon reasonable request.

## Supplementary material

Supplementary Figure 1 FACS gating strategy used during cell isolation. **(A)** Gated by size of the cells. **(B, C)** Exclusion of aggregated cells. **(D)** Gated for sorting of B220 and IgM double positive cells.

Supplementary Figure 2 Similarity of VH segments in the IgM BCR repertoire of each individual. In two BALB/c mice, a specific VH segment was detected at high frequency; therefore, this segment (IGHV1-14) was excluded, and similarity between strains was reanalyzed in the same manner as in **Figure 6**. **(A)** Pairwise similarity between individuals was calculated using the Jaccard index. Values range from 0 to 1, with higher values indicating greater similarity. **(B)** To visualize similarity, distances between samples were calculated using the Jaccard index and dimensionality reduction was performed using t-SNE. Shorter distances between plots indicate higher similarity. **(C)** A bar graph was generated from the Jaccard similarity values calculated in **(A)** to evaluate similarity within and between strains. Results are presented as mean ± SD, and statistical significance between groups is indicated at p < 0.05.
